# What may surprise a rhinologist in everyday clinical practice: silent sinus syndrome or pneumosinus dilatans/pneumocele? Literature review and own experience

**DOI:** 10.1007/s00405-022-07697-w

**Published:** 2022-10-18

**Authors:** Grażyna Stryjewska-Makuch, Magdalena Kokoszka, Karolina Goroszkiewicz, Olga Karłowska-Bijak, Bogdan Kolebacz, Maciej Misiołek

**Affiliations:** 1 Department of Laryngology and Laryngological Oncology, Leszek Giec Upper-Silesian Medical Centre of the Silesian Medical University, Katowice, Poland; 2 Department of Paediatric Otolaryngology, Head and Neck Surgery, Department of Paediatric Surgery, Upper Silesian Child Health Centre, School of Medicine in Katowice, Medical University of Silesia in Katowice, Katowice, Poland; 3 Department of Otorhinolaryngology and Laryngological Oncology in Zabrze, Medical University of Silesia, Katowice, Poland

**Keywords:** Silent sinus syndrome, Pneumocele, Pneumosinus dilatans, Frontal sinus, Maxillary sinus

## Abstract

**Background:**

The aim of the study was to present rare sinus syndromes known as silent sinus syndrome (SSS) and frontal sinus syndrome with excessive pneumatization and bone defects in the wall (pneumocele). The available literature describing pneumocele cases was reviewed.

**Methodology:**

PubMed and Science Direct databases were searched by two independent reviewers. The primary outcome was finding descriptions of the sinus pneumocele. In the end, papers on frontal sinus pneumocele that was not the result of trauma, congenital defects or comorbidities were selected. Moreover, the authors presented their own cases of SSS and pneumocele.

**Results:**

Twelve case reports of frontal sinus pneumocele were found, one own case was presented. In addition, 8 subjects with SSS, diagnosed and treated in the period from September 2017 to May 2022, were described.

**Conclusions:**

With the increasing number of patients suffering from sinus diseases and the growing number of endoscopic surgeries, the knowledge of rare sinus syndromes will increase the safety of the procedures performed.

## Introduction

Rare and seemingly completely different syndromes, namely, silent sinus syndrome (SSS) and pneumosinus dilatans (PD), can confuse even an experienced rhinologist. What they have in common is the lack of inflammatory lesions in the sinuses in imaging tests and no clinical symptoms until visual disturbances or facial deformities occur. In the case of silent sinus syndrome, it is caused by lower orbital wall lowering, and in the case of pneumosinus dilatans, by pushing down the eyeball by the gas-distended frontal sinus. This happens when symptoms concern the maxillary sinus (in SSS) and the frontal sinus (in PD). The process of diagnosing the syndromes becomes even more complicated when the disease affects other sinuses. Silent sinus syndrome is characterized by spontaneous enophthalmos and hypoglobus on the ipsilateral hypoplastic maxilla, secondarily causing orbital floor lowering. The silent sinus is sometimes described as hypoplastic. However, this term should be reserved for congenital unilateral or bilateral underdevelopment. A CT image in sinus hypoplasia is stable, whereas in SSS, changes in CT scans are progressing [1]. SSS can be diagnosed among both children [2, 3] and adults, with a slight predominance between the 3rd and 5th decade[4]. The pathophysiology of SSS remains uncertain. It is suggested that idiopathic obstruction of the ostiomeatal complex results in sinus hypoventilation and discharge. The resorption of secretion leads to the generation of subatmospheric pressure in the maxillary antrum causing maxillary sinus collapse [3, 5–8]. Only a few cases of silent frontal or ethmoid sinuses have been described in the literature [9, 10].

At the other extreme of ventilation disorders, the sinuses are excessively distended with gases, crossing the bone boundaries with or without their destruction.

Pneumosinus Dilatans was first reported in 1898 by Meyes, and the term PD was introduced by Benjamin in 1918, who described an excessively enlarged paranasal sinus filled with air and covered with unchanged mucosa [11]. In 2017, Ricci [12] provided a review of 117 papers describing 134 PD cases. Most often, the process concerned the frontal sinus (63%) followed by the sphenoid sinus (25%); the remaining cases related to the maxillary and ethmoid sinuses. In 84% of the cases, the process involved one sinus. Men between the ages of 20 and 40 suffered from the disease more often, but there were also cases at the age of 12–72 [13–15].

The term pneumosinus dilatans multiplex appears in the literature describing cases of involvement of more than one sinus with excessive aeration of the mastoid process or the frontal and ethmoid sinus complicated by meningocele and sphenoid wing PD associated with a huge arachnoid cyst [16].

Urken introduced the following nomenclature for excessively enlarged frontal sinuses [17]:

1. hypersinus—enlarged sinus with normal walls not extending beyond the boundary of the frontal bone,
2. pneumosinus dilatans—excessively enlarged frontal sinus with a raised front and/or back wall, and a thinner bone without defects,
3. pneumocele—the walls of the distended sinus cross the border of the frontal sinus with a partial bone loss. This kind of changes intrigued the authors of the study the most.

The condition for diagnosing pneumocele is proper aeration without inflammatory lesions in the paranasal sinuses, trauma, or previous surgical interventions. The aetiology of the disease is unknown. Theoretically, ball-valve mechanism, fibro-osseous dysregulation, draining mucocele, genetic predisposition with hormonal trigger during puberty, gas forming bacteria, hormonal dysregulation of bone metabolism are taken into consideration [12]. Unfortunately, there is no evidence for unequivocal confirmation of any of the mechanisms. The fact is that the sinus is over-distended with air, with the mucosa unchanged and the lack of an obvious cause in the structure of the sinus and its ostium. This is in opposition to SSS, where the sinus ostium is closed by drawing the uncinate process into the medial wall of the sinus filled with mucous secretions, gradual absorption of which causes the suction of the sinus walls. In the case of SSS within the maxillary sinus and PD in the frontal sinus (which is the most common), patients do not report symptoms characteristic of chronic rhinosinusitis (CRS) according to the definition of the European Position Paper on Rhinosinusitis and Nasal Polyps 2020 (EPOS 2020) [18]. The problem appears relatively late, when craniofacial deformity or visual disturbances occur as a consequence of eyeball lowering. Rarely do patients report local headaches, pressure in the area of the affected sinus. This might also be the reason for such low recognition of the disease.

The aim of the authors of the study was to review the literature to investigate the frequency of extreme distension of the paranasal sinuses with bone loss, especially in the frontal sinus. Data on gender, age, previous sinusitis or head injuries, comorbidities, and, above all, further treatment of patients diagnosed with pneumocele were investigated.

The authors of the study present their own experiences with SSS of the maxillary sinuses and describe the case of frontal sinus pneumocele, radiological documentation and the adopted surgical strategy.

## Materials and methods

The systematic review was performed according to the PRISMA 2020 checklist [19]. Electronic databases Pubmed and Science Direct were searched from March 1st 2022 until May 1st 2022. Two reviewers (M.K., K.G.) independently executed the comprehensive literature search. The following free-text terms were used for the database search: pneumosinus dilatans, pneumocele, frontal sinus. Although no year limitation was imposed, only studies published in English were considered for inclusion. The search was restricted to humans only. Neither informed consent nor ethics approval was required as this study was a systemic review of published papers.

The study selection process was executed independently by two reviewers (M.K. and K.G.). The criteria for the inclusion and exclusion of the studies in this review were defined.

The inclusion criteria were as follows: (1) case report, (2) diagnosed frontal sinus pneumocele, (3) pneumocele frontalis not caused by head trauma, congenital defects or other diseases. Studies with a putative but unconfirmed link between a head injury and the presence of pneumocele frontalis were included. There was no sex or age restriction.

The exclusion criteria were as follows: (1) patients with pneumocele of the nasal sinuses other than the frontal ones, (2) patients with pneumocele of the frontal sinuses caused by a traumatic brain injury, congenital defects or other diseases, (3) no case report, (4) no access to the full-text paper. Non-English publications were also excluded.

Two authors (M.K and K.G) independently extracted the relevant data from the included studies. When there was insufficient information for extraction or imputation, the corresponding author of that study was contacted for more information. Problems or conflicts during screening and extraction were resolved by discussion among the authors or with the final decision made by the main author (G.S-M).

The following data were recorded from each of the eligible studies: first author, year of publication, sex, age, CRS symptoms, history of head trauma, comorbidities, associated complications, treatment, follow-up.

The risk of bias within the included studies was evaluated by two authors (M.K and K.G.) using the Quality Assessment of Diagnostic Accuracy Studies-2 (QUADAS-2) tool, assessing possible bias in four different categories: patient selection, index test, reference standard, and flow and timing [20].

The authors of the study also performed a literature review focusing on SSS, which was presented in a previously published study [21]. In the period from September 2017 to May 2022, 1766 patients (742 females and 1024 males) with paranasal sinus diseases were admitted and operated on at the Department of Laryngology and Laryngological Oncology in Katowice–Ochojec. SSS patients were selected from this group.

## Results

**Fig. 1:**
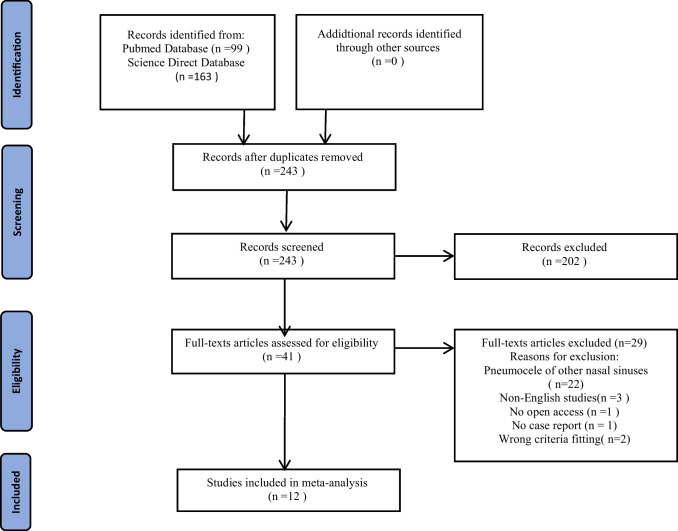
Literature search strategy

A total of 262 studies were retrieved through the systematic search of bibliographic databases. After removing 19 duplicates, 243 papers were eligible for title and abstract screening**.** After title and abstract screening, the authors excluded 243 articles and read the full text of 41 papers. In the end, 12 publications containing case reports of 13 patients with pneumocele of the frontal sinuses were included in meta-analysis. 22 papers were rejected due to the presence of pneumocele in the paranasal sinuses other than the frontal ones, 3 papers were not available in English, in 2 studies, the described cases were proven to be related to a head injury or congenital defect, 1 publication did not contain a case report, and 1 had no open access. The selection process is presented in Fig. 1. The risk of bias for all studies was assessed using the Quality Assessment of Diagnostic Accuracy Studies-2 (QUADAS-2) tool [20].

All 12 studies were written in English and published from 1974 to 2018. Of the 13 patients, the majority were male (11 men and 2 women). The mean age was 37.7 years, the oldest was a 67-year-old woman and the youngest was an 18-year-old man. All patients were diagnosed with pneumocele of the frontal sinuses. One person had pneumocele of the frontal and sphenoidal sinuses simultaneously.

1 subject suffered a head injury 21 years before, but as the authors add “the relationship between head trauma and the frontal pneumocele is not clear”. 4 out of 13 patients had symptoms of chronic sinusitis. Only 3 patients had additional conditions: (1) dilated sphenoidal sinuses, orbital and frontal subcutaneous aerocele, (2) type III frontal recess air cell and (3) respiratory epithelial adenomatoid hamartoma with nasal polyps. Complications caused by pneumocele developed in seven patients. Two were complicated by pneumocephalus. Ocular complications occurred in four patients (orbital roof defect, orbital emphysema, proptosis, hypoglobus and orbital swelling). In all but one of the cases, surgery was the recommended method of treatment. Four patients underwent endoscopic surgery and three refused to undergo the surgical procedure.

The subjects whose health status was observed after accepting (or refusing) treatment, showed complete or partial regression of symptoms caused by pneumocele in the frontal sinuses.

The characteristics of the twelve included studies are summarised in Table 1.

**Table 1:** Study characteristics

	Papers	Lead Author [ref] (year)	Sex	Age	Chronic Sinusitis Symptoms	Head Trauma History	Comorbidies	Associated complications	Treatment	Follow-up
1	Abnormally large frontal sinus. II. Nomenclature, pathology, and symptoms	Urken ML et al. [17] (1987)	Male	33	No	None	None	None	Surgery refused by the patient	None
			Male	36	No	None	None	None	Osteoplastic flap procedure with fat obliteration of the sinus	None
2	Pneumocele vs. pneumosinus dilatans: review of the literature with a case of frontal sinus pneumocele	Acar M. et al. [42] (2004)	Male	23	No	None	None	None	Surgery refused by the patient	None
3	Enlargement of Frontal Sinus, Case Report	Andejani D. et al. [43] (2018)	Male	26	Yes	None	None	None	FESS and Open Reshaping of the Anterior Table	No evident recurrence
4	Frontal Sinus Pneumocele Associated with Respiratory Epithelial Adenomatoid Hamartoma and Nasal Polyps	Gu Z. et al. [44] (2011)	Male	32	Yes	None	Respiratory Epithelial Adenomatoid Hamartoma, Nasal Polyps	None	Endoscopic Sinus Surgery	Postoperative headache disappearance
5	Spontaneous pneumocephalus associated with pneumocele of the frontal sinus	Park CY. et al. [37] (2010)	Male	35	No	None	None	Pneumocephalus	Conservative Treatment	Complete resolution of symptoms and pneumocephalus
6	Pneumocele of the frontal sinus producing orbital roof defect: case report and review of literature	Abdel-Aal AK. et al. [36] (2008)	Female	31	Yes	None	Type III frontal recess air cell	Orbital roof defect	Large left frontal endoscopic sinusotomy and resection of the obstructing air cells; frontal sinus stent insertion	None
7	A case of Orbital Emphysema Associated with Frontal Sinus Pneumocele	Sasaki T. et al. [39] (2013)	Female	67	No	None	None	Orbital Emphysema	Left Frontal Craniotomy	No appearance of an orbital emphysema
8	Post-traumatic Pneumocele of the Frontal Sinus	Karadag D. et al. [45] (2008)	Male	46	No	Head trauma 21 years prior*	None	None	Surgery refused by the patient	None
9	Pneumocele—A rare Cause of Air in the Orbit	Boulos PR. et al. [46] (2004)	Male	52	Yes	Nasal fracture at age 7	None	Proptosis and Hypoglobus	Anterior Orbitotomy and Endoscopic sinusotomy	Decrease of the exophthalmos
10	Surgical Correction of an Extensive Skull Base Defect as a Result of Pneumocele	Ramprasad VH et al. [35] (2016)	Male	54	No	None	None	Pneumocephalus	48-h bed rest Surgery: the posterior table defect reconstruction with a pericranial flap	No recurrence of pneumocephalus
11	Pneumocele of the frontal and sphenoidal sinuses	Jarvis JF. et al. [47] (1974)	Male	18	No	None	Dilated sphenoidal sinuses, Orbital and Frontal Subcutaneous Aerocele	Orbital swelling	Surgery: external Frontoethmoidectomy Incision, Opening into the Nose and its Drainage	No sign of swelling
12	Frontal sinus pneumocele	Eskandary H. et al.[48] (1999)	Male	37	No	None	None	Frontal bossing	Surgery: the removal of the anterior wall of the frontal sinus	3 years, no complication, appropriate contour of forehead

*Authors add that relationship between head trauma and the frontal pneumocele is not clear

**Fig. 2:**
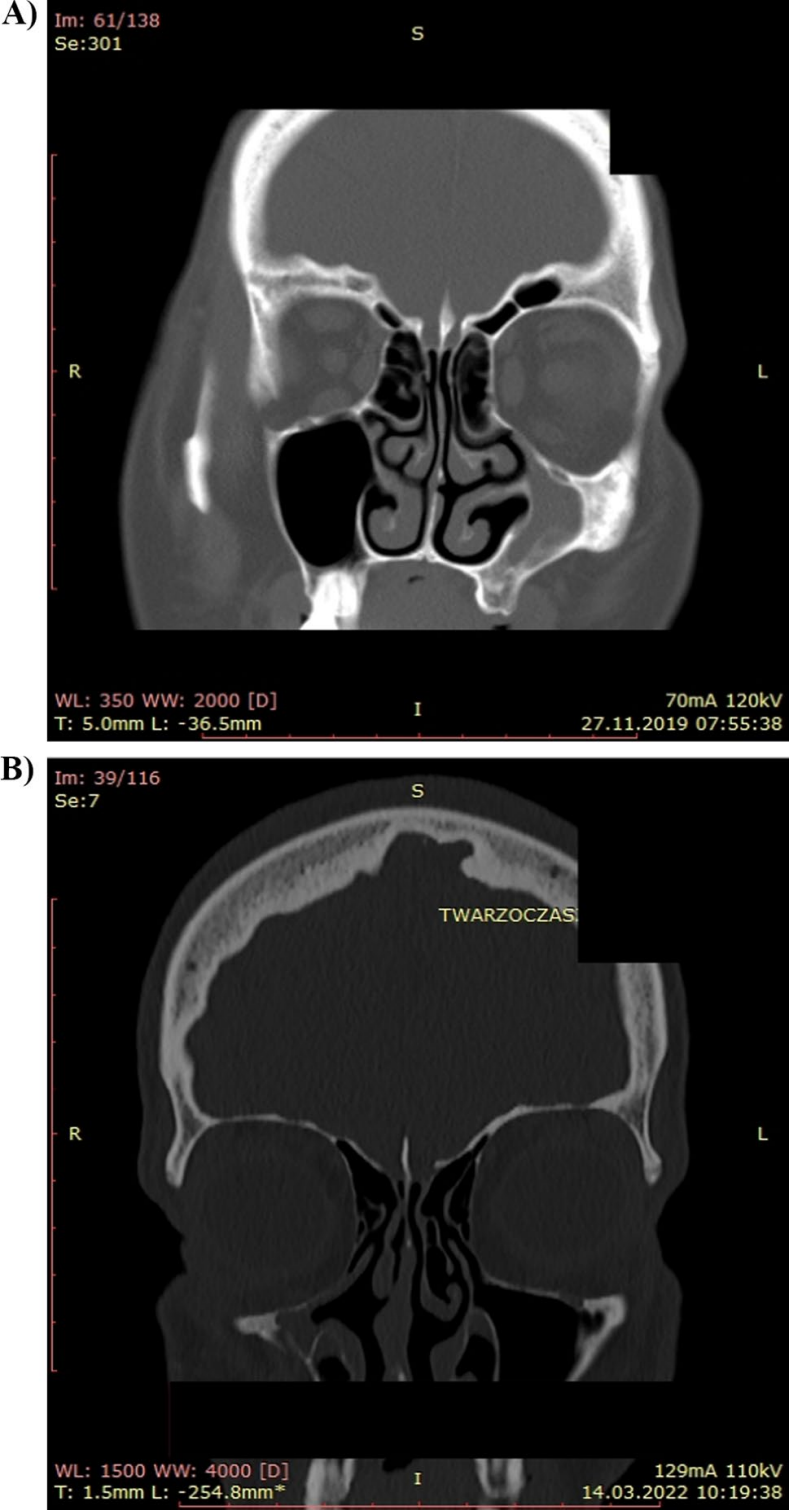
CT images, **A**–**B** show the silent sinus syndrome involving the maxillary sinus. **A** 58-year-old female with SSS of the left maxillary sinus. **B** 40-year-old female with SSS of the right maxillary sinus

The silent sinus syndrome was diagnosed in 8 patients, 4 men and 4 women, The mean age of diagnosis was 45.4 years (the youngest patient was 31, the oldest was 75) (Fig. 2A, B).

In the patients with SSS, the problem concerned the maxillary sinuses. All the patients were treated endoscopically, each time anteroethmidectomy of the diseased maxillary sinus was performed, ensuring its proper drainage.

In March 2022, a 32-year-old man was admitted to the Department of Laryngology and Laryngological Oncology in Katowice–Ochojec, reporting tissue bulging in the right frontal area. The skin on the forehead was pale and painless. With stronger pressure, defects were felt in the scale of the frontal bone. The patient reported headaches when the ambient temperature changed. Since 2017, he had had frequent sinus infections in the fall and winter period. In October 2019, he noticed a concavity of tissues in the glabella area sized 2 × 2 cm.

After the secretion was blown from the nasal cavity, there was swelling of the lower eyelid of the right eye. On September 20, 2020, the patient was qualified for plastic surgery of the septum. The operation was uneventful. After the procedure, at the beginning of 2020, there was swelling in the frontal area that was gradually increasing.

The patient did not report any comorbidities, including atopy. Outpatient diagnostics for microvasculitis did not confirm the disease.

**Fig. 3:**
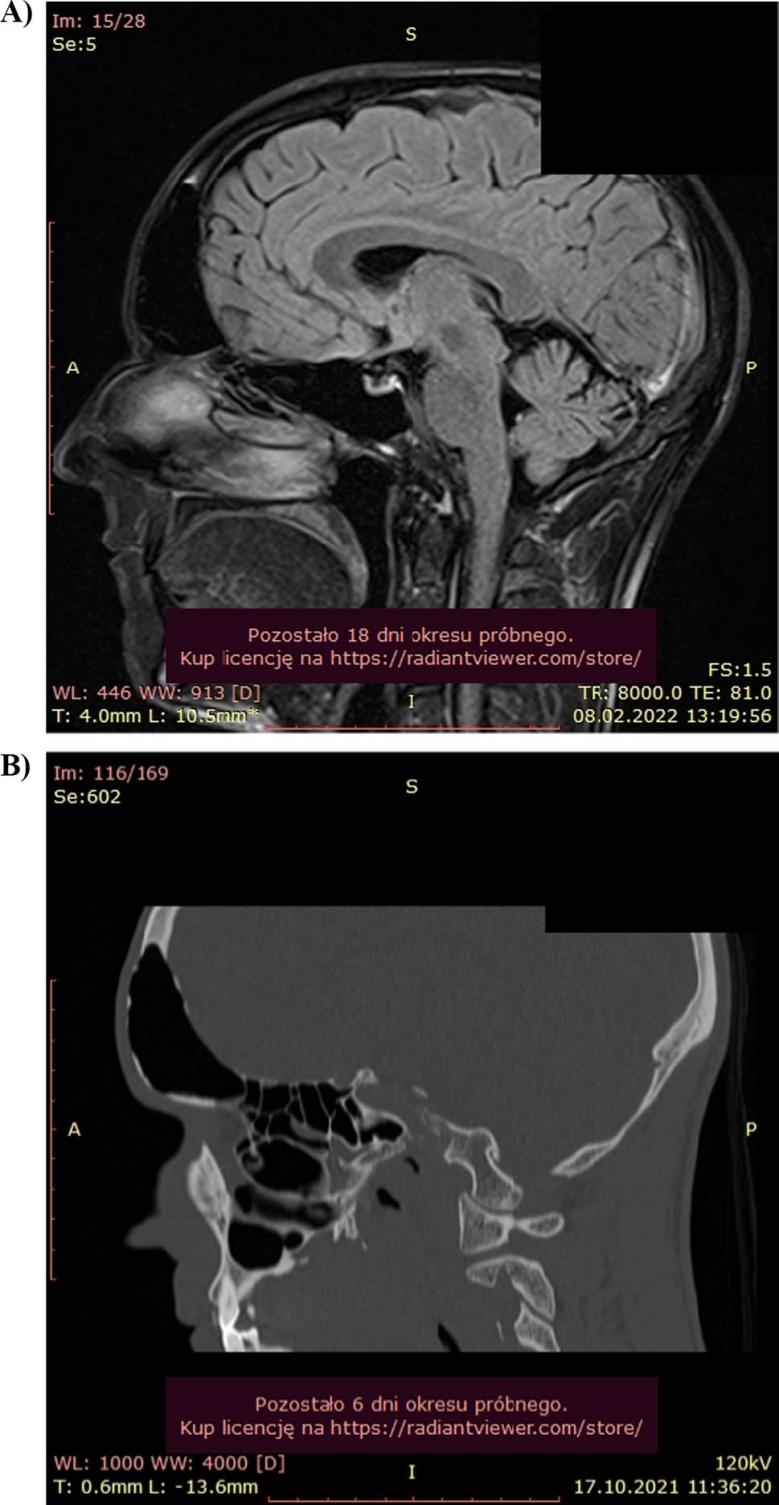
MRI (**A**) and CT (**B**) images show the enlargement of the frontal sinus with bone thining

CT and MRI showed defects in the anterior and posterior walls of the frontal sinus (Fig. 3 A, B). The airiness of the paranasal sinuses and the patency of the ostiomeatal complexes were preserved. Bacteriological examination of the nasal cavity revealed the growth of the physiological bacterial flora and Candida fungi. Endoscopic examination of the nasal cavity showed no abnormalities. Pneumocele was diagnosed and the patient was offered endoscopic surgical treatment consisting in widening the right frontal sinus ostium. Until the publication preparation, the patient had not consented to surgery.

On August 16th 2022, after obtaining the patient’s consent, a decision was made to perform frontal sinus ostial dilatation. Bilateral balloon sinuplasty was performed under general anesthesia, under negative pressure, under control of the navigation (NuVent EM Sinus Dilatation System, Frontal 6 × 17 mm, Medtronic). During the procedure, after decontamination of the nasal vestibule skin, bacteriological material was collected on both sides from the middle nasal meatus for culture examination in aerobic and anaerobic conditions. The patient’s condition after the surgery was very good, he did not report any nasal bleeding or pain. Methicillin-susceptible *Staphylococcus epidermidis* was isolated in all four bacteriological studies. The patient was instructed to stay under constant laryngological observation.

## Discussion

The authors of the study present rare diseases involving deformation of the paranasal sinuses, which, if incorrectly diagnosed, may surprise the surgeon and lead to postoperative complications. The literature review suggests that pneumocele not caused by head trauma or comorbidities is rare. So far, the cause of the disease has not been identified and no treatment guidelines have been developed. Our literature review found 12 papers describing 13 patients with pneumocele of the frontal sinus. In only three cases, endoscopic sinus surgery was sufficient, the remaining cases required osteotomy and reconstruction of the damaged sinus wall. Two cases were complicated by pneumocephalus, and three involved orbital lesions.

Pneumocele does not give symptoms for a long time, hence perhaps the reason for such a rare diagnosis of the disease. Similarly, in the early stage of SSS, the patient does not report any symptoms. In the material collected by the authors, the silent sinus syndrome was in all cases accompanied by inflammatory lesions in other sinuses and the patients manifested symptoms typical of CRS, which facilitated the diagnosis of SSS. In SSS, without facial deformity and diplopia, treatment consists in restoring proper drainage of the diseased sinus [22, 23]. When the disease is advanced, reconstruction of the superior wall of the maxillary sinus is considered [24–28].

It is much more difficult to deal with excessive distension of the frontal sinus, PD and pneumocele. The unknown cause of gas accumulation in the sinus translates to difficulties in making a treatment decision. In the case of bone deformity of the scales of the frontal bone in PD, the anterior table of the frontal sinus is removed.

The bone fragment can be secured with low profile titanium mini-plates [29–32].

Patel et al. presented surgical management of the excessively distended frontal sinus (without local defects) depending on the patency of the nasofrontal duct, recommending endoscopic restoration of function in the absence of a cosmetic defect. In contrast, frontal bossing requires an open procedure [33–35]. After correcting the shape of the frontal bone scales, the sinus mucosa is removed by filling the sinus lumen with autogenous bone, adipose tissue or hydroxyapatite.

Due to its rarity, there are no set treatment standards for pneumocele. There are reports of endoscopic widening of the frontal sinus ostium without disturbing the continuity of the mucosa and removing the bony partitions separating the frontal recess from supraorbital cells, intersinus septal cells, and frontal recess cells [36, 37]. Similarly, the authors of the study proposed endoscopic surgery to widen the frontal sinus ostium.

The limitation of our observations was the low recognition of SSS and pneumocele in the daily clinical practice of an ENT specialist and a short period of observation of patients after surgery. The presented cases of rare diseases of the paranasal sinuses may surprise every rhinologist performing endoscopic operations. It should be noted that in approximately 25% of patients with symptomatic frontal sinus pneumocele having comorbid intracranial pathology, the most common is meningioma [38–40].

Paranasal sinus diseases affect about 10.9% of the European population [41], the number of operations is increasing, and therefore, the chance of encountering SSS or PD in everyday practice is real.

## Data Availability

The data sets used and/or analysed during the current study are either included in this published article or are available from the corresponding author on reasonable request.

## Abbreviations

SSS: Silent sinus syndrome
PD: Pneumosinus dilatans
CT: Computed tomography
CRS: Chronic rhinosinusitis
MRI: Magnetic resonance imaging
